# Simultaneous Determination of 32 Pyrrolizidine Alkaloids in Two Traditional Chinese Medicine Preparations by UPLC-MS/MS

**DOI:** 10.1155/2022/7611501

**Published:** 2022-09-14

**Authors:** Shi Cheng, Wei Sun, Xiaoning Zhao, Ping Wang, Wensheng Zhang, Shunnan Zhang, Xiangwei Chang, Zhengliang Ye

**Affiliations:** ^1^School of Chinese Materia Medica, Tianjin University of Traditional Chinese Medicine, Tianjin 300193, China; ^2^International Industry Center, Tasly Pharmaceutical Group Co. Ltd., Tianjin 300410, China; ^3^College of Pharmacy, Anhui University of Chinese Medicine, Hefei 230012, China; ^4^Anhui Province Key Laboratory of Pharmaceutical Preparation Technology and Application, Hefei 230012, China; ^5^Engineering Technology Research Center of Modernized Pharmaceutics, Anhui Education Department (AUCM), Hefei 230012, China

## Abstract

Pyrrolizidine alkaloids (PAs) constitute a class of phytotoxin which demonstrates strong hepatotoxicity. In China, many plants containing PAs are used as traditional medicines or medicinal preparations, which could harm human health and safety. Xiaoyao Tablet (XYT) is an antidepressant drug registered in the European Union (EU), Compound Danshen Dropping Pills (CDDP) is a commonly used drug for coronary heart disease, and phase III clinical study is ongoing in the United States. The purpose of this study is to provide data to support the use of Chinese medicine preparations internationally and to establish analytical methods for 32 PAs in XYT and CDDP. The extraction parameters that were optimized include solid-phase extraction (SPE) cartridge, extraction method, and extraction solvent. Then ultra-performance liquid chromatography coupled with triple-quadrupole linear ion-traptandem mass spectrometry (UPLC-MS/MS) was developed to effectively and efficiently quantify the 32 PAs of the XYT and CDDP. The analytical methods for XYT and CDDP were verified respectively. For XYT, the analytical method for 32 PAs was linear, and the correlation coefficient *r* was greater than 0.994; the recovery (REC%) at 10–2000 *μ*g/kg was 73.3%–118.5%, and the relative standard deviation (RSD%) was 2.1%–15.4%. The CDDP REC% was 71.8%–112.0%, and the RSD% was 2.0%–17.1%. This study provides technical and data support for the registration of Chinese patented medicines in the EU, controls quality and ensures safety, and is committed to the internationalization and standardization of Chinese patented medicines.

## 1. Introduction

PAs and their corresponding N-oxides (PANOs) are a large group of phytotoxins which cause hepatotoxicity [1]. At present, more than 660 pyrrolidine nuclear alkaloids and their corresponding nitrogen oxide forms have been identified. PAs are mainly distributed in the *Compositae*, the *Leguminosae,* and the *Boraginaceae* families and mainly exist in plants in the form of nitrogen oxides. In China, many plants containing PAs are used as traditional medicines or medicinal preparations, which could harm human health and safety, such as *Tussilago farfara* and *Arnebia euchroma* [2]. Animal-derived food such as honey [3, 4], milk [5], eggs [6], and animal organs is prone to contain PAs. PAs are metabolized by the human body to produce unsaturated dehydropyrrole metabolites, which can be complexed with large molecules such as proteins, causing liver cell necrosis, and liver toxicity [7].

In order to control the intake of PAs, relevant departments have successively proposed regulations and recommendations. In 2016, the European Medicines Agency (EMA) clearly stipulated that at least 28 PAs of Chinese herbal medicine should be controlled, and the limit of PAs should be ≤ 0.35 *μ*g PAs/day, and it was recommended to adopt the article “Determination of Pyrrolizidine Alkaloids in Plant Material by SPE-LC-MS/MS (BfR-PA-Tea-2.0/2014)” in the determination of PAs in Chinese herbal medicine by MS [8]. In 2018, the EMA updated the “Guideline on Quality of Herbal Medicinal Products/Traditional Herbal Medicinal Products” and the “Guideline on Specifications: Test Procedures and Acceptance Criteria for Herbal Substances, Herbal Preparations and Herbal Medicinal Products/Traditional Herbal Medicinal Products”. The released drafts have added new requirements for the control of PAs in medicinal materials and extracts [9, 10] where College ter Beoordeling van Geneesmiddelen (CBG) requires that the maximum daily dose of drugs' PAs and their nitrogen oxides has to be controlled below 0.35 *μ*g/day/50 kg. In Germany, Bundesinstitut Für Risikobewertung (BfR) used SPE and liquid chromatography-tandem mass spectrometry (LC-MS/MS) to determine 28 PAs in tea and tea products and 17 PAs in honey [11, 12]. According to the *Chinese Pharmacopoeia* 2020, the content of adonifurin in *Senecionis Scandentis Hebra* should be not more than 0.004% [13]. In 2021, the EMA adopted new regulations specifying the control of 28 PAs [14]. The most common test method for PAs at present is based on LC-MS/MS. On the one hand, tandem mass spectrometry (MS/MS) has a high sensitivity for limit of detection (LOD), and it can detect PAs which are low in content in some plants [15]; on the other hand, multiple PAs/PANOs with similar structures cannot be simultaneously separated by ultra-performance liquid chromatography (UPLC) separation method, and mass spectrometry is required to confirm their ion fragments for qualitative and quantitative determination of multiple PAs. PAs are alkaloids, which are highly hydrophilic, and they are generally extracted using 0.1% formic acid-methanol [16], 0.1% formic acid, 95% ethanol, and other solvents. Purification of the honey is mostly done using SPE and matrix dispersed solid-phase extraction (MSPD), such as strong cation-exchange solid-phase extraction cartridge (SCX) and strong cation-exchange solid-phase extraction cartridge (PCX) [17], and good recovery (REC%) can be obtained.

Xiaoyao tablet (XYT) [18] is a typical modern Chinese patented medicine, and it is also registered with the EMA. It is derived from the “Taiping Huimin Heji Ju Prescription” in the Song Dynasty, which is a representative prescription for soothing the liver and regulating “qi”. It is clinically used to treat the liver “qi” discomfort, the liver “qi” stagnation, and other diseases, including depression, gynecological diseases, etc. It is a large compound containing multiple medicinal materials with complex chemical components composed of *Atractylodis Macrocephalae Rhizoma*, *Paeoniae Radix Alba*, *Angelicae Sinensis Radix*, *Bupleuri Radix,* and *Glycyrrhizae Radix Et Rhizoma Praeparata Cum Melle*. CDDP [19] is a modern and innovative traditional Chinese medicine compound, which is composed of *Salviae Miltiorrhizae Radix Et Rhizoma*, *Notoginseng Radix Et Rhizoma,* and *Borneolum*. It is mainly used for the prevention and treatment of cardiovascular diseases. The CDDP is currently undergoing a phase III clinical study at the Food and Drug Administration (FDA).

At present, the detection of PAs is mostly concentrated in the raw plants of traditional Chinese medicine [20, 21] and honey. There is no standard method designed specifically for the identification and quantification of the alkaloids in traditional Chinese medicine preparations. This study focuses on the quantitative methods for the PAs of two traditional Chinese medicine preparations marketed internationally to ensure that they meet the guidelines for international product safety risks. Based on comprehensive literature and regulatory guidelines [22, 23], this paper studied the 32 PAs based on multiple regulatory requirements, comprehensive experimental research, and the availability of reference substances. However, due to the characteristics of PAs, the test involves multiple indicators, low limits, and complex matrices. Ultra-performance liquid chromatography coupled with triple-quadrupole linear ion-trap tandem mass spectrometry (UPLC-MS/MS), which is highly specific and sensitive, is used in this study to combine chromatographic separation and mass spectrometry and pass the solid phase at the same time. The purposes are to ensure that the extraction method eliminates the interference of the sample matrix and to analyze the 32 PAs quantitatively.

## 2. Materials and Methods

### 2.1. Chemicals and Materials

Methanol (LC-MS grade) was purchased from Merck (Darmstadt, Germany); formic acid and ammonium formate (chromatography grade) were obtained from Sigma-Aldrich Corporation (St. Louis, MO, USA); ammonia and sulfuric acid were both of analytical grade; five SPE cartridges including SCX, PCX, X-C, C18, and C18/SCX were all 500 mg/6 mL and from Agela Technologies (Tianjin, China); ultrapure water was obtained from a Milli-Q water purification system (Millipore, Billerica, MA, USA).

### 2.2. Preparation of Standard Solutions

**Figure 1:**
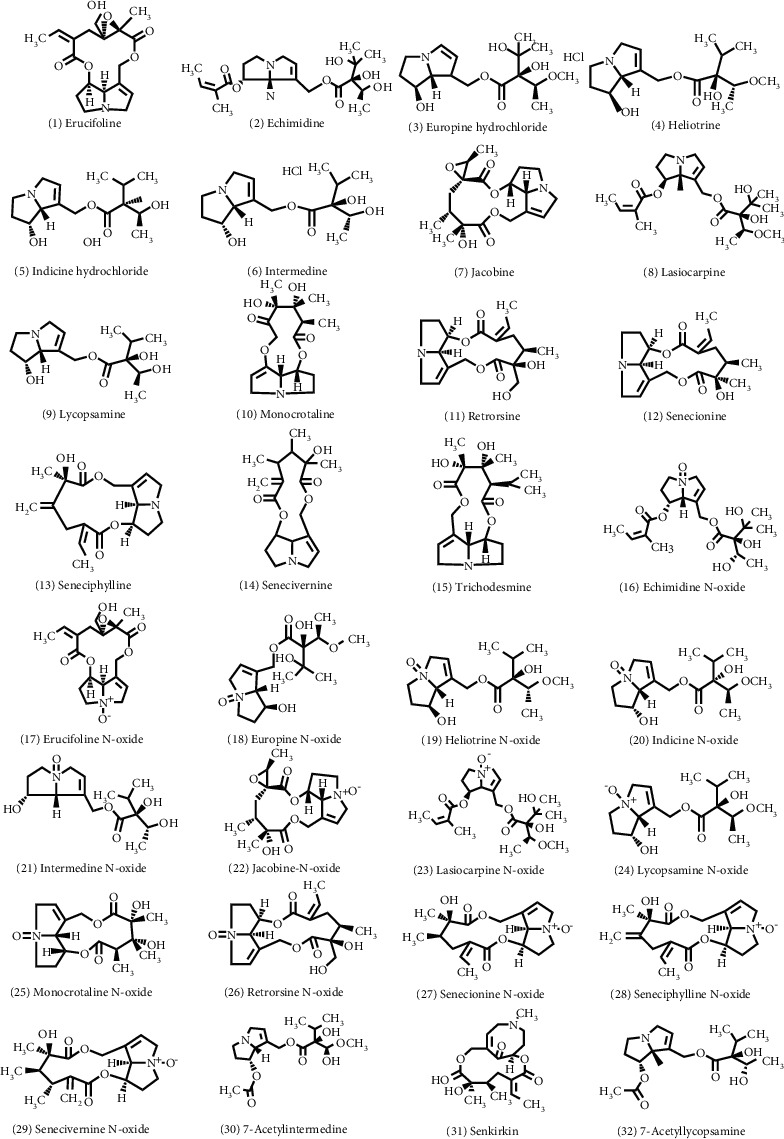
Molecular structural formulas of 32 PAs and their N-oxides.

The following PA standards were purchased from Phytolab Laboratories (Vestenbergsgreuth, Germany): echimidine (Em), erucifoline (Er), europine hydrochloride (Eu), heliotrine (He), indicine hydrochloride (Ic), intermedine (Im), jacobine (Jb), lasiocarpine (Lc), lycopsamine (Ly), monocrotaline (Mc), retrorsine (Re), senkirkine (Sk), senecionine (Sn), seneciphylline (Sp), senecivernine (Sv), trichodesmine (Td), 7-acetyllycopsamine (7-Ly), 7-acetylintermedine (7-Im), echimidine-N-oxide (EmNO), erucifoline-N-oxide (ErNO), europine-N-oxide (EuNO), heliotrine-N-oxide (HeNO), indicine-N-oxide (IcNO), intermedine-N-oxide (ImNO), jacobine-N-oxide (JbNO), lasiocarpine-N-oxide (LcNO), lycopsamine-N-oxide (LyNO), monocrotaline-N-oxide (McNO), retrorsine-N-oxide (ReNO), senecionine-N-oxide (SnNO), seneciphylline-N-oxide (SpNO), and senecivernine-N-oxide (SvNO). Of all the standards, the 7-Im, 7-Ly, Ic, and IcNO were tested with the above references, while the remaining 28 were tested by the EMA. The molecular formulas of 32 PAs/PANOs are shown in Figure 1.

Five milligram of each PAs was accurately weighed and dissolved in methanol. Moreover, among all the standards available, IcNO, McNO, and ReNO were dissolved in 50% methanol to prepare a single reference stock solution (*c* = 200 mg/mL). Based on the different instrument response of each substance, 32 types of PAs single reference stock solutions of 50 *μ*L (20 *μ*L–200 *μ*L, the specific volume is adjusted according to the final concentration) were accurately measured, transferred into a 10 mL volumetric flask, diluted to volume with 50% methanol, stirred, and used as a mixed reference stock solution for XYT (standard solution 2). The mixed reference solution for CDDP (standard solution (2) was prepared in the same way as standard solution 1, not including IcNO, ImNO, LyNO, and ReNO. The mixed reference solution for CDDP (standard solution 3) was prepared in the same way as standard solution 1, just including IcNo, ImNo, LyNo, and ReNo. The specific concentration of the reference substance is shown in Table 1. All reference solutions were stored at −4°C and protected from light.

### 2.3. Sample Collection and Preparation

XYT, its two extracts, and CDDP were from Tasly Pharmaceutical Group Co., Ltd. (Tianjin, China), and *Atractylodis Macrocephalae Rhizoma* and other medicinal materials were from Zhongtian Pharmaceutical Co. Ltd. (Gansu, China). The substances and their batch numbers are listed as follows: XYT (batch number: 20190101); extract 1 (batch number: 20190407); extract 2 (batch number: 20190504); *Atractylodis Macrocephalae Rhizoma* (batch number: 201903201); *Paeoniae Radix Alba* (batch number: 201903201); *Angelicae Sinensis Radix* (batch number: 1903202); *Bupleuri Radix* (batch number: 201903201); *Glycyrrhizae Radix Et Rhizoma Praeparata Cum Melle* (batch number: 201903203); and CDDP (batch number: 20190104). The reserved samples were stored in the International Industry Center of Tasly Pharmaceutical Group Co. Ltd.

XYT was crushed into powder. The capsule of CDDP was removed, and the content was taken 2.0 g of the test product which was accurately weighed; then, 40 mL of 1% formic acid was added and sonication was performed at room temperature for 20 minutes (min). Then it was centrifuged at 5000 rpm for 10 min. The supernatant was then transferred to a 50 mL volumetric flask and diluted to volume. Then, 25 mL of supernatant was precisely pipetted and applied to the PCX cartridge. Then, it was pretreated with 5 mL of methanol and 5 mL of 0.05 M sulfuric acid solution and rinsed with 5 mL of 0.05 M sulfuric acid solution and 10 mL of methanol. The PAs were washed with 10 mL ammonia/methanol (1 : 3, V/V) solution, evaporated, and finally reconstituted with 50% methanol to a total volume of 5 mL. The test product was taken and a blank matrix solution was prepared in the same way as the preparation method of the test solution.

### 2.4. Chromatographic Conditions

In this study, the UPLC-MS/MS 8060 (SIL-30AC, LC-30AD, CTO-20AC, Shimadzu, Japan), Thermo Hypersil Gold C18 (100 mm × 2.1 mm, 1.9 *μ*m) chromatographic column was used. 0.05% formic acid solution (containing 2.5 mM/L ammonium formate) was used as mobile phase A, and methanol-0.05% formic acid solution (containing 2.5 mM/L ammonium formate) was used as mobile phase B. The elution gradient was 0 min, 95%A, 0 min–0.5 min, and then it was changed with a linear gradient to 90%A, 0.5 min–5 min, 85%A, 5 min–11 min, 80%A, 11 min–13 min, 35%A, 13 min–14 min, 5%A, 14 min–16.5 min, keeping isocratic elution for 2.5 min, 16.5 min–16.6 min, 95%A, 16.6 min–18 min, 95%A. The flow rate was set at 0.4 mL/min, the column temperature was maintained at 40°C, and the injection volume was 2 *μ*L.

The mass spectrometry conditions were as follows: electrospray ionization (ESI) was used as the ion source, positive ion scanning mode was used, and the monitoring mode was multiple reaction monitoring (MRM). The atomizing, drying, and heating gas flow were set at 3.0 L/min, 10.0 L/min, and 10.0 L/min, respectively, with an interface, DL, and heating block temperature of 300°C, 250°C, and 400°C respectively and CID gas of 270 kPa. The detection and quantification of the substances were performed using MRM with the MassLynx™ version 4.1 software (Waters). The retention time, ion pairs, and collision energy (CE) were summarized in Table 1.

In this paper, matrix-matched calibration is used for quantification. The extract of the blank sample is used as the diluent, and solutions of different concentrations are added to prepare the solutions, so that they all have the same ionization conditions, so as to reduce the interference of the matrix on the results. 2 *μ*L of the reference solution and the test solution were accurately drawn respectively for determination, and the contents of PAs were calculated by the standard curve method.

### 2.5. Method Validation

The method was validated in house by evaluating calibration curves, specificity, LOD, LOQ, precision, stability, and recovery.

#### 2.5.1. Calibration Curves, Specificity, LOD, and LOQ

Calibration curves were plotted with peak areas versus their corresponding concentrations of the reference substance. An appropriate amount of the mixed reference solution was precisely drawn and added to the blank matrix solution to prepare a linearity reference. Then, the linearity reference solution was accurately drawn and sampled for quantitative analysis. Two values were entered for each concentration and the corresponding peak area of each was recorded. The prepared concentration of the solution was taken as the *x*-axis, while the mean of the two peak areas measured was taken as the *y*-axis. A six-point calibration was used to plot the standard curve and calculate the *r* value.

In the specificity test, the interference peak response was less than 5% of the LOQ response of the spiked sample at the retention time of the target peak.

For LOD and LOQ, reference substances were added to blank samples since matrix-matched calibrations were used. LOD and LOQ were determined at a signal-to-noise ratio (S/N) of about 3 and 10, respectively.

#### 2.5.2. Precision and Stability

The same batch of samples were obtained and spiked with an intermediate level (100 *μ*g/kg) of the mixed reference solution to evaluate the precision of the developed method. Based on the preparation method of the test solution, two analysts prepared 6 spiked sample solutions in parallel and calculated the REC% and RSD% of 32 PAs in the 6 samples.

In order to evaluate the stability of the method, the same batch of samples was obtained and added to the mixed standard with an intermediate level (100 *μ*g/kg). Samples were then prepared according to the preparation method of the test sample and stored in the refrigerator (−15–25°C). The REC% of 32 PAs was analyzed at 0 h, 24 h, and 48 h respectively, and the RSD% of REC% at three points was calculated.

#### 2.5.3. Recovery and Repeatability

Moreover, to evaluate the recovery and repeatability of the method, the method of adding a reference substance to the sample to calculate the REC% and RSD% was adopted. Since the 32 PAs have different responses on the UPLC-MS/MS, standard solutions of different concentrations were prepared. Among which the spiked levels of Em, Eu, He, Im, Ic, Lc, Ly, LcNo, 7-Im, 7-Ly, IcNo, and ImNo were 10 *μ*g/kg, 40 *μ*g/kg, and 200 *μ*g/kg. The spiked levels of Er, Jb, Mc, Sp, Td, Sk, ErNo, EuNo, HeNo, JbNo, McNo, SpNo, and LyNo were 25 *μ*g/kg, 100 *μ*g/kg, and 500 *μ*g/kg. The spiked levels of Re, Sv, Sn, EmNo, SvNo, and SnNo were 50 *μ*g/kg, 200 *μ*g/kg, and 1000 *μ*g/kg. The spiked levels of ReNo were 100 *μ*g/kg, 400 *μ*g/kg, and 2000 *μ*g/kg. Three replicates were prepared for each level, and the REC% and RSD% of 9 spiked samples were calculated. Each standard was spiked with three different levels, 3 samples were prepared for each level, and the REC% and RSD% of the 9 spiked samples were calculated.

#### 2.5.4. Matrix Effect

Matrix effect was defined as ion suppression or enhancement in the process of substance ionization. The matrix effect was evaluated using the slope of calibration curves of standards in solvent and matrix-matched solutions [24].

## 3. Results and Discussion

### 3.1. Development of the LC-MS/MS Method

**Figure 2:**
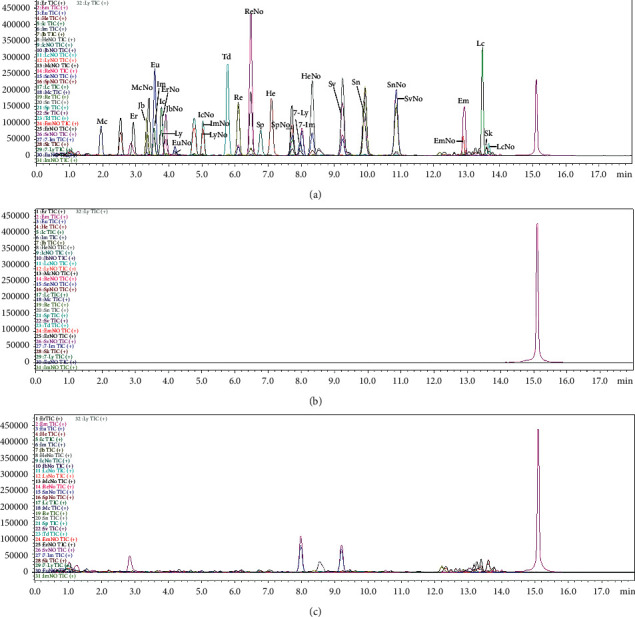
UPLC-MS/MS chromatogram of XYT: (a) 50 ppb standard solution (mixed with XYT blank solution), (b) XYT blank solvent, and (c) XYT blank solution. For the abbreviations of the compounds, see 2.1 Standards.

The chromatographic conditions such as the mobile phase and the elution gradient were studied in the preliminary study to achieve a desirable chromatographic profile with optimized retention time and peak profiles. The chromatographic method of reference [17] was optimized, and the separation results of 32 PAs after optimization are shown in Figure 2. 0.05% formic acid in water (containing 2.5 mM/L ammonium formate) and methanol-0.05% formic acid solution (containing 2.5 mM/L ammonium formate) were used as mobile phase A to optimize the gradient elution, and the separation effect was found to be adequate. Compared with the commonly used 0.1% formic acid in water and 0.1% formic acid-acetonitrile solution, the separation effect was found to be better, as isomers Ly, Ic, IcNo, and ImNo which were previously not separated could achieve total separation as LyNo and IcNo (resolution = 1.73).

Thirty-two types of PAs reference substance mixed solutions were analyzed. Firstly, the flow injection method was used to perform a full scan of the precursor ions in positive ion mode to determine the molecular ion peak, and then the molecular ion of the test compound was identified as the parent ion to perform a full scan of its product ions. Two characteristic product ions were selected where the ion pair with a high S/N, good peak shape, and low interference is selected as the quantitative ion pair. MRM positive ion mode was used to optimize various mass spectrometry parameters. The best mass spectrometry parameters were obtained and the separation effect of 32 PAs was observed to determine the retention time of each substance.

### 3.2. Development of the Sample Extraction and Cleanup

**Figure 3:**
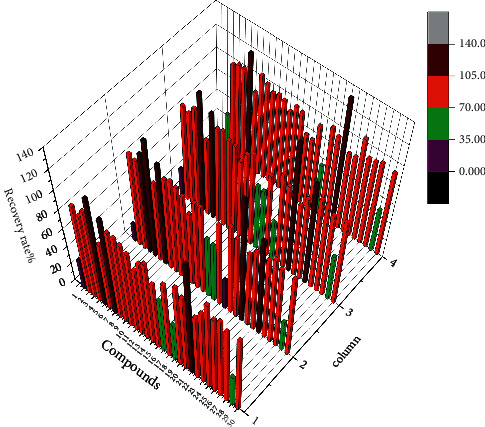
PCX, SCX, X-C, C18/SCX SPE cartridges for the recovery of PAs. *X*-axis: **1**. Em, **2**. Er, **3**. Eu, **4**. He, **5**. Ic, **6**. Im, **7**. Jb, **8**. Lc, **9**. Mc, **10**. Re, **11**. Sk, **12**. Sn, **13**. Sp, **14**. Sv, **15**. Td, **16**.7-Ly, **17**.7-Im, **18**. EmNO, **19**. ErNO, **20**. EuNO, **21**. HeNO, **22**. IcNO, **23**. JbNO, **24**. LcNO, **25**. LyNO, **26**. McNO, **27**. ReNO, **28**. SnNO, **29**. SpNO, **30**. SvNO. *Y*-axis: **1**. PCX, **2**. SCX, **3**. X-C, **4**. C18/SCX. For the abbreviations of the compounds, see Section 2.1 Standards.

In order to optimize the PA/PANO separation, the extraction and cleanup procedures were developed. In LC-MS/MS determination, purification methods such as SPE are often used to reduce matrix interference and improve accuracy. In the selection of SPE cartridge, the purification effects of 5 SPE cartridges, PCX, SCX, X-C, C18, and C18/SCX, were investigated. Among all the cartridges studied, the retention effect of the C18 cartridge was found to be extremely poor. The liquid using the X-C cartridge was not easily filtered and had poor retention of Er, ErNo, 7-Im, and 7-Ly, and the REC% was 30.2%–69.2%. Moreover, the SCX cartridge had poor retention effect on Er, ErNo, SnNo, 7-Im, and 7-Ly, and the REC% was 20.0%–68.5%. In addition, the C18/SCX cartridge had poor retention effect on Er, ErNo, and SnNo, and the REC% was 41.9%–53.1%. The PCX, SCX, X-C, C18, C18/SCX cartridges all did not retain ImNO and Ly. After extraction and purification optimization, the PCX cartridge was able to recover the 32 PAs studied. Therefore, the PCX cartridge was finally used for enrichment and purification, as shown in Figure 3.

**Figure 4:**
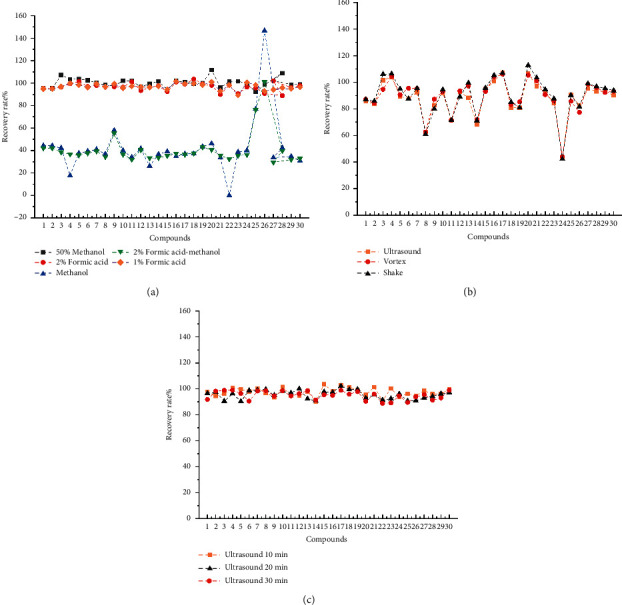
Xiaoyao tablet preparation extraction method, extraction time, and extraction solvent selection results. (a) Extraction solvent selection results, (b) extraction method selection results, and (c) extraction time selection results. The numbering of the 30 target analytes in Figure 4 is same as in Figure 3.

For the selection of extraction solvent, extraction method, and extraction time, the following series of studies were carried out. In order to optimize the extraction solvent selection, five extraction solvents, including methanol, 50% methanol, 2% formic acid solution, 1% formic acid solution, and 2% formic acid-methanol, were selected for study. The results showed that the extraction effects of methanol and 2% formic acid-methanol on the reference substance were both poor. On the other hand, for the solution with 50% methanol, 2% formic acid solution, and 1% formic acid solution, each had no significant difference in the extraction effect of the reference substance respectively. However, since the solution with 50% methanol had poor peak shape and the solution with 2% formic acid-water was more acidic, 1% formic acid-water solution was used as the final extraction solvent. A few selections of extraction methods such as ultrasonic, shake, and vortexing were compared. The REC% of the reference substance under the three extraction conditions (20 min sonication, 20 min shaking, and 20 min vortexing) were studied, respectively. The results showed that there was no significant difference in the REC% of the reference substance under the three extraction conditions. Finally, sonication was selected as it is easy to operate. After determining the extraction method as sonication, the effect of different sonication times on recovery was compared. There was no significant difference in the REC% of 32 PA reference substances after sonication for 10 min, 20 min, and 30 min. It was found that sonication for 20 minutes was enough to extract PAs, so an intermediate level of sonication time was used for extraction, as shown in Figure 4.

**Figure 5:**
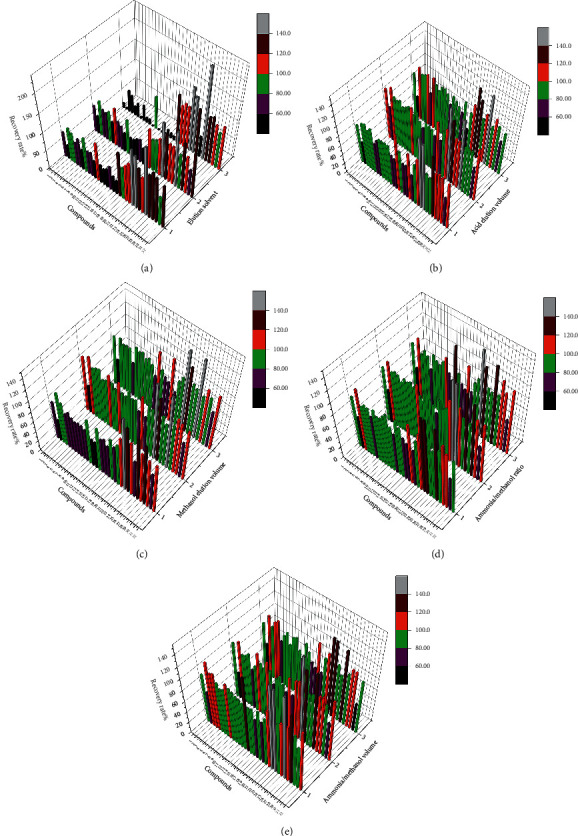
Selection results of CDDP preparation pretreatment formula. (a) Elution solvent selection result. (b) Acid elution volume selection result. (c) Methanol elution volume selection result. (d) Ammonia/methanol elution ratio selection result. (e) Ammonia/methanol elution volume selection result. *X*-axis: **1**. Em, **2**. Er, **3**. Eu, **4**. He, **5**. Ic, **6**. Im, **7**. Jb, **8**. Lc, **9**. Ly, **10**. Mc, **11**. Re, **12**. Sk, **13**. Sn, **14**. Sp, **15**. Sv, **16**. Td, **17**.7-Ly, **18**.7-Im, **19**. EmNO, **20**. ErNO, **21**. EuNO, **22**. HeNO, **23**. IcNO, **24**. ImNO, **25**. JbNO, **26**. LcNO, **27**. LyNO, **28**. McNO, **29**. ReNO, **30**. SnNO, **31**. SpNO, **32**. SvNO.

After the cartridge was identified, the elution cleanup step for the cartridge was optimized. Based on comprehensive studies and literature, three elution solvents have been studied, including 5 mL of 0.05 M sulfuric acid aqueous solution with 10 mL of methanol (solvent 1), 5 mL of water with 10 mL of methanol (solvent 2) and 15 mL of water (solvent 3). It was found that the impurity removal effect of (solvent 3) was poor, and the matrix interfered greatly with the sample recovery. The REC% of (solvent 1) was significantly higher than that of (solvent 2). In addition, 5 mL of 0.05 M sulfuric acid aqueous solution with 10 mL of methanol was in line with the theory of alkaloid acid extraction and alkali precipitation and could fully extract alkaloids in the sample. For satisfactory elution power, 5 mL of 0.05 M sulfuric acid aqueous solution with 10 mL of methanol was selected as the elution solvent. The REC% is shown in Figure 5(a).

Next, further studies were performed on the volume of elution solvent, so various volumes of the elution solvent (0.05 M sulfuric acid aqueous solution with methanol) were studied. Three different volumes (3 mL, 5 mL, and 8 mL) were examined for 0.05 M sulfuric acid aqueous solution, and three different volumes (5 mL, 10 mL, and 15 mL) were examined for methanol. The results showed that the different volumes of the elution solvent had no significant effect on the REC%, so an intermediate volume was selected as the volume of elution solvent, 5 mL of 0.05 M sulfuric acid aqueous solution with 10 mL of methanol, for rinsing to remove impurities. The REC% is shown in Figures 5(b) and 5(c).

Then, the ratio and volume of the elution solvent were optimized, and the effects of different elution ratios and volumes of ammonia/methanol on the recoveries of PAs were studied. Three different ammonia/methanol ratios (3 : 15, 3 : 12, and 3 : 9) were studied. The results showed that the REC% of ammonia/methanol (3 : 9) was the highest among the three ratios. Three different volumes of ammonia water/methanol (1 : 3, V/V) (5 mL, 10 mL and 15 mL) were studied. The results showed that 10 mL of ammonia water/methanol (1 : 3, V/V) had the highest elution ability, as shown in Figures 5(d) and 5(e).

### 3.3. Selection of Standard Adding Method

Preliminarily determined conditions were used to confirm the REC% of the PAs. As a result, it was found that the REC% of IcNO, ImNO, LyNO, and ReNO was significantly higher (close to 150%), and further tests were performed to eliminate the unstable factors of the sample. In the references and review of the operation process, there may be the phenomenon that Ic, Im, Ly, and Re are converted into IcNO, ImNO, LyNO, and ReNO. The results showed that the single standard REC% of IcNO, ImNO, and LyNO test solution and the REC% of IcNO, ImNO, LyNO, and ReNO in the four mixed standard test solutions and the 30 mixed standard test solutions (except IcNO, ImNO, LyNO, and ReNO) were at a normal level. In addition, it was found that 30 mixed standard test products (except IcNO, ImNO, LyNO, and ReNO) showed chromatographic peaks in the IcNO, ImNO, and LyNO channels, while another 30 mixed reference solutions did not show any chromatographic peaks. For CDDP, the REC% of IcNO, ImNO, LyNO, and ReNO in the sample solution was all at a normal level. The results showed that the sample treatment process or the CDDP polyethylene glycol excipient might have caused the conversion of the above substances. Therefore, in order to ensure the accuracy of the test results and reduce the interference of the matrix, 28 reference substances and four substances were added to the matrix of the CDDP for study.

### 3.4. Effect of Polyethylene Glycol on Recovery

In terms of the recovery test, when reference substances were added, polyethylene glycol excipients may have interfered with the conversion of Ic, Im, Ly, and Re into IcNO, ImNO, LyNO, and ReNO. In the study where reference substances were added separately, the REC% of Er and ErNo was below 60%, and it was observed that polyethylene glycol affected the enrichment and elution process of the alkaloids. Polyethylene glycol is a high-molecular polymer with a chemical formula of HO (CH_2_CH_2_O)_n_H [25]. It has excellent dispersibility and adhesion profile. However, the mechanism of how polyethylene glycol promotes the conversion of PAs to PANOs is unknown and further studies are required.

### 3.5. Method Verification for XYT

For calibration curves, the correlation coefficient *r* is greater than 0.994. The standard curve, linear range, and *r* are shown in Table 2. For specificity study, the interfering peak responses were all less than 5% of the LOQ responses of the spiked samples at the retention time of the target peak. The LOD of XYT was 0.1–1.7 *μ*g/kg, and the LOQ of all matrices was 0.2 *μ*g/kg–7.2 *μ*g/kg (refer to Table 3).

The results showed that the intermediate precision was acceptable, and the RSD% was 1.3–16.5%. The specific values are shown in Table 4. In terms of stability, the results showed that the 32 PAs of XYT were stable within 48 hours, of which 28 PAs had RSD% of less than 10% and RSD% of 1.9% (IcNO)–16.2% (JbNO).

For repeatability and accuracy, the mean REC% of the eight different matrices was 73.3%–118.5%, and the RSD% was 2.1%–15.4%. The specific values are shown in Table 5. Both methods had adequate repeatability and accuracy, the REC% was within acceptable range, and the repeatability met the requirements.

### 3.6. Method Verification for CDDP

The verification method of the CDDP is the same as that of Section 2.5 where reference solutions 2 and 3 were also included when the reference substance was added. The results are shown in Table 6. For accuracy, the REC% of the 32 PAs in the sample was 71.8–112.0%, and the RSD% was 2.0–17.1%.

### 3.7. Analysis of the Necessity for Detection

The main taxonomic group containing the PAs includes *Boraginaceae*, *Senecio,* and *Eupatorium* of *Asteraceae* and *Crotalaria* of *Leguminosae* [26, 27]. The 32 PAs were not detected in the XYT and the CDDP. The XYT only contains *Atractylodis Macrocephalae Rhizoma*, *Paeoniae Radix Alba,* and other medicinal materials, while the CDDP contains *Salviae Miltiorrhizae Radix Et Rhizoma* and other medicinal materials that are not of the above-mentioned principal taxonomic category, which is consistent with other literature [28, 29]. Since the plants studied may have the risk of contamination with the other plants containing PAs during the harvesting process, the measures taken to control the PAs in the medicines in this paper are able to reduce the workload and ensure the quality and safety of the medicines. At present, there are few types of PA detection methods, which are able to detect approximately seven to eight types of alkaloids, most of which are concentrated in honey, Chinese medicine, and tea [30, 31]. However, the detection method of PAs in Chinese medicine preparations has not been reported. In 2008, EMA recommended that Chinese herbal medicines should be tested for the presence of PAs. Subsequently in year 2022, the latest guidelines have been issued, which requires all Chinese patented medicines registered in the EU to submit the test results for PAs. For Chinese patented medicines which are composed of a variety of traditional Chinese medicines, the accurate detection of the presence of PAs faces great matrix interference.

## 4. Conclusion

In summary, by extraction with formic acid solution, purification with PCX cartridge, detection with UPLC-MS/MS, and quantification with external standard method, the content of the 32 PAs in XYT and CDDP was determined, and the method was suitable for the detection of the components of the XYT and the other two intermediate extracts. This study established the relevant detection method for traditional Chinese medicine preparations which have complex matrices. It has high sensitivity but simple operation. It also provides a reference for the detection of samples with complex matrices and is of great significance in ensuring the quality and safety of drugs. In this paper, a detection method for PAs of two international products (XYT and CDDP) was successfully developed. It has major significance as these two drugs are registered internationally. This study demonstrated a breakthrough in the limited detection of the Chinese patented medicines.

## Figures and Tables

**Table 1:** Retention time, concentration, and mass spectrometry conditions for 32 PAs.

Compound^1^	Retention time^2^ (min)	Concentration (*μ*g/mL)	Quantitative transition			Qualitative transition		
			Precursor ion	Product ion	CE^3^	Precursor ion	Product ion	CE
Em	13.04	0.3926	398.1	120.1	32	398.1	149.0	30
Er	2.90	1.0325	350.1	120.1	37	350.1	138.1	37
Eu	3.94	0.4048	330.1	138.1	30	330.1	156.2	40
He	7.56	0.4088	314.2	138.1	30	314.2	156.2	35
Ic	4.13	0.3958	300.2	156.2	40	300.2	138.1	30
Im	3.89	0.4128	300.2	156.2	40	300.2	138.1	30
Jb	3.68	1.0135	352.2	120.0	35	352.2	155.1	38
Lc	13.56	0.4038	412.1	120.1	35	412.1	138.1	40
Ly	4.13	0.4118	300.2	138.1	10	300.2	156.2	10
Mc	2.21	1.0300	326.0	138.1	40	326.0	120.1	45
Re	6.50	2.0300	352.2	120.1	40	352.2	138.1	40
Sk	13.50	1.0045	366.1	168.0	9	366.1	150.0	9
Sn	10.44	1.9540	336.2	120.1	38	336.2	138.1	38
Sp	7.20	1.0120	334.3	120.1	38	334.3	138.1	38
Sv	9.77	2.0300	336.3	120.1	41	336.3	138.2	41
Td	6.17	0.9880	354.3	120.1	45	354.3	222.3	38
7-Ly	8.41	0.4200	342.2	120.1	9	342.2	198.1	9
7-Im	8.15	0.4098	342.3	120.1	10	342.3	198.2	10
EmNO	13.00	2.0680	414.3	220.1	42	414.3	352.0	35
ErNO	3.74	1.0085	366.1	118.1	42	366.1	120.1	42
EuNO	4.54	1.0085	346.3	172.1	8	346.3	111.1	8
HeNO	8.81	1.0270	330.2	172.2	40	330.2	111.1	55
IcNO	5.15	0.3958	316.2	172.1	38	316.2	138.2	39
ImNO	5.15	0.4240	316.2	172.1	6	316.2	138.0	6
JbNO	4.24	0.9975	368.3	120.2	45	368.3	296.3	35
LcNO	13.70	0.3936	428.2	254.2	40	428.2	137.2	45
LyNO	5.15	0.9670	316.2	172.1	40	316.2	138.0	40
McNO	3.25	1.0310	342.1	118.1	50	342.1	137.1	40
ReNO	6.86	4.0980	368.1	118.1	45	368.1	136.0	40
SnNO	11.41	2.0230	352.2	118.1	40	352.2	136.0	45
SpNO	8.18	0.9735	350.2	120.1	42	350.2	138.1	38
SvNO	10.43	2.0210	352.2	118.1	42	352.2	136.1	42

^1^ For the abbreviations of the compounds, refer to Section 2.1 (Standards). ^2^ Determined using the UPLC conditions. ^3^ CE: collision energy.

**Table 2:** Standard curve, linear range, and *R* for PAs in the XYT.

Compound^1^	Standard curve line	Linear range (*μ*g/mL)	*r * ^2^	Compound	Standard curve line	Linear range (*μ*g/mL)	*r*
Em	*y* = 103074*x* + 11930.1	0.7852 – 39.26	0.999	7-Ly	*y* = 15161.2*x* + 2419.04	0.84 – 42	0.999
Er	*y* = 23539.7*x* + 36563.2	2.065 – 103.25	0.999	7-Im	*y* = 34526.6*x* − 785.171	0.8196 – 40.98	0.999
Eu	*y* = 137290*x* + 16650.4	0.8096 – 40.48	0.995	EmNO	*y* = 3897.03*x* + 1680.78	4.136 – 206.8	0.999
He	*y* = 117780*x* + 22207.8	0.8176 – 40.88	0.999	ErNO	*y* = 29337.1*x* + 11196.1	2.017 – 100.85	0.994
Ic	*y* = 91690.0*x* + 15739.7	0.7916 – 39.58	0.998	EuNO	*y* = 4361.23*x* + 1906.23	1.984 – 99.2	0.998
Im	*y* = 45160.1*x* + 5115.68	0.8256 – 41.28	0.999	HeNO	*y* = 84714.4*x* + 14931.2	2.054 – 102.7	0.998
Jb	*y* = 18459.2*x* + 371.455	2.027 – 101.35	0.998	IcNO	*y* = 18459.2*x* + 371.455	0.7916 – 39.58	0.998
Lc	*y* = 213437*x* + 17634.3	0.8076 – 40.38	0.994	ImNO	*y* = 5658.26*x* + 23.7988	0.848 – 42.4	0.995
Ly	*y* = 42618.1*x* + 34005.6	0.8236 – 41.18	0.998	JbNO	*y* = 23061.7*x* − 7354.04	1.995 – 99.75	0.998
Mc	*y* = 37794.5*x* + 18435.9	2.06 – 103	0.997	LcNO	*y* = 15720.3*x* + 6353.57	0.7872 – 39.36	0.996
Re	*y* = 11762.0*x* + 336.356	4.06 – 203	0.999	LyNO	*y* = 11762.0*x* + 336.356	1.934 – 96.7	0.999
Sk	*y* = 2559.25*x* + 4467.82	2.009 – 100.45	0.994	McNO	*y* = 17940.2*x* − 3716.42	2.062 – 103.1	0.999
Sn	*y* = 24934.7*x* − 1030.49	3.908 – 195.4	0.999	ReNO	*y* = 12647.5*x* − 8885.25	8.196 – 204.9	0.996
Sp	*y* = 16347.7*x* + 4728.13	2.024 – 101.2	0.997	SnNO	*y* = 29089.1*x* + 2805.59	4.046 – 202.3	0.999
Sv	*y* = 20077.5*x* − 2234.22	4.06 – 203	0.999	SpNO	*y* = 28269.4*x* − 4078.85	1.947 – 97.35	0.998
Td	*y* = 43131.0*x* + 1767.45	1.976 – 98.8	0.999	SvNO	*y* = 33290.3*x* + 13311.5	4.042 – 202.1	0.999

^1^ For the abbreviations of the compounds, see 2.1 Standards. ^2^ Correlation coefficient.

**Table 3:** LODs and LOQs of PAs in various matrices.

Compound	XYT		Extract 1	Extract 2	Atractylodis Macrocephalae Rhizoma	Paeoniae Radix Alba	Bupleuri Radix	Angelicae Sinensis Radix	Glycyrrhizae Radix Et Rhizoma Praeparata Cum Melle
	LOD1 (*μ*g/kg)	LOQ2 (*μ*g/kg)	LOQ (*μ*g/kg)	LOQ (*μ*g/kg)	LOQ (*μ*g/kg)	LOQ (*μ*g/kg)	LOQ (*μ*g/kg)	LOQ (*μ*g/kg)	LOQ (*μ*g/kg)
Em	0.2	0.6	0.4	0.4	0.6	0.7	0.7	0.7	0.6
Er	0.3	1.5	1.2	1.3	1.7	1.7	1.8	1.3	1.1
Eu	0.2	0.5	0.4	0.5	0.2	0.2	0.7	0.2	0.2
He	0.1	0.5	0.2	0.3	0.3	0.4	0.7	0.5	0.4
Ic	0.2	0.6	0.6	0.5	0.3	0.3	0.7	0.7	0.5
Im	0.2	0.7	0.2	0.5	0.2	0.3	0.8	0.5	0.4
Jb	0.2	1.2	1.5	1.4	0.9	1.0	1.9	0.7	1.0
Lc	0.1	0.6	0.2	0.2	0.4	0.2	0.7	0.5	0.4
Ly	0.1	0.5	0.3	0.3	0.4	0.3	0.6	0.2	0.3
Mc	0.3	1.1	0.9	0.7	1.1	1.3	1.7	0.5	1.1
Re	1.2	3.2	2.0	1.8	3.0	2.2	3.5	2.1	2.3
Sk	0.6	1.5	1.3	1.7	1.5	0.9	1.8	1.9	1.2
Sn	0.7	2.4	1.7	1.7	3.2	3.3	3.5	2.7	2.1
Sp	0.4	1.7	0.9	0.8	1.2	2.0	2.0	1.1	1.3
Sv	1.0	3.4	1.7	2.2	3.7	3.0	3.4	2.4	2.1
Td	0.3	1.8	1.6	1.2	1.8	1.2	1.5	1.1	1.1
7-Ly	0.1	0.6	0.3	0.3	0.4	0.3	0.7	0.4	0.3
7-Im	0.1	0.3	0.4	0.5	0.5	0.4	0.6	0.2	0.2
EmNO	1.0	3.7	2.2	2.8	3.0	2.7	3.1	3.4	2.7
ErNO	0.7	1.9	1.8	0.9	1.2	1.2	1.7	0.8	1.0
EuNO	0.5	1.7	1.7	1.2	0.7	1.5	1.2	1.5	1.2
HeNO	0.3	1.4	1.7	1.3	1.5	2.0	1.8	1.8	1.2
IcNO	0.2	0.7	0.7	0.6	0.4	0.5	0.6	0.2	0.4
ImNO	0.2	0.5	0.4	0.6	0.3	0.3	0.5	0.3	0.4
JbNO	0.4	1.6	1.5	1.6	0.9	1.8	1.5	1.2	0.7
LcNO	0.1	0.8	0.4	0.4	0.5	0.3	0.6	0.3	0.3
LyNO	0.3	1.7	1.1	1.4	0.7	1.1	1.5	1.5	0.9
McNO	0.4	1.5	1.7	1.1	1.4	1.5	1.3	0.8	1.0
ReNO	1.7	6.4	4.4	5.6	3.3	6.8	6.8	5.5	7.2
SnNO	0.9	3.2	3.6	2.5	2.6	1.9	3.3	2.6	1.9
SpNO	0.4	1.9	1.8	1.8	1.1	0.9	1.6	1.3	1.1
SvNO	0.8	2.9	3.2	1.9	2.4	3.2	2.0	2.7	3.5

^1^ LOD: limit of detection. ^2^ LOQ: limit of quantification.

**Table 4:** Mean recoveries and precision of the method for PAs in various matrices.

Compound	Average recovery rate (%) at intermediate level ± RSD (%)（each *n* = 6 samples）							
	XYT	Extract 1	Extract 2	Atractylodis Macrocephalae Rhizoma	Paeoniae Radix Alba	Bupleuri Radix	Angelicae Sinensis Radix	Glycyrrhizae Radix Et Rhizoma Praeparata Cum Melle
Em	103.0 ± 9.9	99.9 ± 6.4	104.0 ± 7.2	89.3 ± 8.7	81.9 ± 5.2	108.2 ± 8.1	90.7 ± 6.2	91.3 ± 5.8
Er	83.1 ± 6.3	87.3 ± 2.0	94.6 ± 9.6	86.3 ± 2.0	81.8 ± 14.1	88.6 ± 6.5	87.9 ± 5.5	83.7 ± 2.4
Eu	95.5 ± 3.0	85.9 ± 7.8	90.3 ± 8.0	95.1 ± 5.4	94.0 ± 4.1	99.0 ± 4.3	98.2 ± 5.5	89.2 ± 3.0
He	93.6 ± 2.5	91.0 ± 6.5	98.6 ± 4.1	95.7 ± 1.6	97.5 ± 3.6	102.0 ± 4.1	98.1 ± 4.0	96.6 ± 1.8
Ic	92.3 ± 2.6	93.4 ± 8.6	92.5 ± 8.8	96.1 ± 4.0	102.7 ± 3.6	106.5 ± 6.2	95.2 ± 2.4	92.9 ± 10.2
Im	90.6 ± 5.8	96.8 ± 11.4	98.4 ± 6.6	101.9 ± 3.3	110.3 ± 6.0	115.3 ± 6.8	102.0 ± 5.3	97.9 ± 7.7
Jb	87.7 ± 8.2	87.0 ± 3.7	95.3 ± 6.9	92.7 ± 6.8	85.9 ± 13.9	95.4 ± 9.8	84.6 ± 3.2	87.1 ± 4.3
Lc	88.5 ± 8.9	78.7 ± 14.2	91.4 ± 8.3	87.5 ± 7.7	90.1 ± 4.5	95.3 ± 13.1	83.5 ± 7.9	81.7 ± 10.8
Ly	98.7 ± 4.6	99.5 ± 10.5	92.5 ± 9.7	96.5 ± 3.2	102.4 ± 6.5	110.2 ± 8.2	101.0 ± 6.5	82.5 ± 4.6
Mc	83.7 ± 11.9	91.9 ± 11.2	84.6 ± 12.0	90.4 ± 6.6	97.0 ± 9.2	95.3 ± 7.2	92.0 ± 12.4	81.9 ± 5.8
Re	97.1 ± 1.3	90.9 ± 8.1	99.7 ± 8.1	90.6 ± 4.0	96.7 ± 7.5	107.3 ± 4.3	103.3 ± 8.9	93.4 ± 2.9
Sk	100.4 ± 12.7	106.4 ± 8.7	90.6 ± 4.1	99.0 ± 6.9	88.5 ± 8.7	104.9 ± 14.9	85.4 ± 6.0	97.8 ± 14.0
Sn	95.9 ± 4.8	90.2 ± 12.1	106.2 ± 10.9	91.4 ± 5.3	93.2 ± 6.7	100.5 ± 2.9	95.4 ± 3.4	96.7 ± 8.4
Sp	92.8 ± 3.0	86.6 ± 5.1	89.9 ± 8.5	85.8 ± 5.3	90.0 ± 8.8	96.7 ± 9.7	86.3 ± 7.9	86.6 ± 7.0
Sv	92.3 ± 4.6	96.4 ± 4.0	96.2 ± 7.2	91.3 ± 1.8	91.6 ± 7.1	103.1 ± 1.9	93.3 ± 2.1	92.3 ± 5.1
Td	90.1 ± 4.9	85.0 ± 12.1	89.7 ± 8.8	91.0 ± 2.7	95.6 ± 6.8	96.8 ± 2.9	97.4 ± 3.6	90.7 ± 7.0
7-Ly	90.3 ± 5.4	81.4 ± 11.9	76.9 ± 5.7	87.9 ± 3.9	88.0 ± 7.0	83.3 ± 8.1	86.0 ± 10.6	82.3 ± 2.4
7-Im	93.5 ± 2.7	86.7 ± 6.9	83.2 ± 9.6	96.9 ± 3.7	85.5 ± 3.5	95.6 ± 13.9	88.1 ± 4.5	83.4 ± 7.1
EmNO	74.6 ± 4.8	88.9 ± 11.8	92.6 ± 7.3	92.1 ± 10.4	95.0 ± 14.2	88.9 ± 12.5	88.8 ± 11.0	95.3 ± 14.9
ErNO	78.5 ± 4.4	106.4 ± 11.4	94.0 ± 8.8	92.7 ± 3.0	102.8 ± 6.1	85.9 ± 8.1	99.5 ± 5.6	90.3 ± 4.4
EuNO	93.5 ± 6.8	96.1 ± 7.6	98.2 ± 14.7	96.6 ± 5.0	104.3 ± 4.5	99.2 ± 7.3	100.0 ± 9.6	94.8 ± 10.0
HeNO	100.1 ± 2.2	100.5 ± 2.9	97.8 ± 4.9	97.8 ± 3.1	101.7 ± 4.3	99.1 ± 1.7	102.5 ± 2.1	98.4 ± 1.8
IcNO	96.0 ± 3.6	106.9 ± 3.7	101.1 ± 10.2	101.7 ± 4.4	105.7 ± 5.3	96.0 ± 4.2	101.0 ± 4.8	98.3 ± 3.1
ImNO	101.7 ± 16.5	99.0 ± 11.5	93.9 ± 11.5	97.4 ± 5.2	103.5 ± 4.4	98.0 ± 5.6	100.1 ± 14.5	98.6 ± 8.7
JbNO	75.6 ± 7.0	107.4 ± 13.3	83.1 ± 11.7	91.7 ± 6.7	103.0 ± 5.6	89.0 ± 6.0	108.1 ± 6.0	94.3 ± 5.6
LcNO	92.4 ± 15.7	98.1 ± 9.2	92.0 ± 6.3	94.2 ± 5.8	93.9 ± 14.6	92.7 ± 13.2	88.0 ± 14.9	78.9 ± 12.9
LyNO	99.0 ± 6.9	111.8 ± 9.9	94.1 ± 12.7	100.1 ± 5.1	109.1 ± 5.4	101.6 ± 3.5	102.1 ± 7.4	97.7 ± 7.1
McNO	85.2 ± 6.5	106.8 ± 8.4	86.5 ± 12.7	94.7 ± 6.2	104.4 ± 9.2	83.9 ± 7.2	96.4 ± 5.0	87.2 ± 11.6
ReNO	96.2 ± 4.4	108.5 ± 7.8	96.4 ± 10.1	103.3 ± 1.8	110.9 ± 4.1	101.5 ± 2.3	105.0 ± 5.4	103.9 ± 3.9
SnNO	94.9 ± 3.9	110.0 ± 7.4	90.1 ± 14.5	104.4 ± 3.6	110.6 ± 5.9	98.7 ± 1.2	96.2 ± 4.8	99.2 ± 6.6
SpNO	76.1 ± 4.0	82.0 ± 8.4	83.9 ± 5.3	83.9 ± 6.9	86.9 ± 10.2	76.6 ± 10.2	80.1 ± 7.7	80.7 ± 8.3
SvNO	92.6 ± 2.4	110.9 ± 7.9	90.3 ± 14.8	101.4 ± 5.8	110.0 ± 5.5	96.2 ± 3.9	92.5 ± 4.4	95.7 ± 6.8

**Table 5:** Mean recoveries and repeatability of the method for PAs in the XYT.

Compound	Average recovery rate at 3 levels of standard addition (%) ± RSD (%) (each *n* = 9 samples)							
	XYT	Extract 1	Extract 2	Atractylodis Macrocephalae Rhizoma	Paeoniae Radix Alba	Bupleuri Radix	Angelicae Sinensis Radix	Glycyrrhizae Radix Et Rhizoma Praeparata Cum Melle
Em	100.9 ± 10.8	95.2 ± 12.2	106.9 ± 8.2	83.3 ± 9.7	79.1 ± 13.9	117.1 ± 4.9	87.1 ± 7.8	90.1 ± 8.8
Er	82.2 ± 8.8	88.1 ± 13.9	94.9 ± 12.3	85.9 ± 2.5	74.6 ± 4.3	100.4 ± 9.8	83.0 ± 6.8	93.9 ± 12.4
Eu	95.8 ± 3.1	89.9 ± 2.8	85.8 ± 9.0	91.7 ± 7.3	95.4 ± 7.4	105.5 ± 8.2	97.4 ± 5.7	92.4 ± 7.1
He	97.0 ± 4.2	94.8 ± 6.2	95.7 ± 7.6	95.0 ± 4.9	96.4 ± 4.2	110.1 ± 6.4	94.4 ± 5.9	98.8 ± 9.3
Ic	96.0 ± 8.7	93.6 ± 10.4	89.2 ± 4.9	97.8 ± 4.1	98.7 ± 4.3	112.5 ± 4.1	96.2 ± 6.9	103.6 ± 9.4
Im	96.4 ± 12.1	97.1 ± 11.5	96.6 ± 9.9	99.7 ± 5.3	103.1 ± 11.8	118.5 ± 2.7	97.6 ± 7.4	100.4 ± 10.0
Jb	84.7 ± 11.6	86.8 ± 11.2	99.4 ± 9.5	88.5 ± 5.5	82.2 ± 9.8	102.3 ± 7.7	80.0 ± 9.7	90.6 ± 12.2
Lc	90.4 ± 11.9	73.8 ± 9.0	87.9 ± 8.4	82.9 ± 8.7	91.8 ± 14.0	111.2 ± 10.1	81.5 ± 10.5	96.8 ± 14.7
Ly	96.5 ± 11.5	96.2 ± 10.7	92.6 ± 11.9	95.6 ± 4.9	99.2 ± 5.7	119.2 ± 3.1	98.4 ± 5.4	91.3 ± 9.7
Mc	80.5 ± 12.0	84.9 ± 12.4	85.7 ± 14.3	86.1 ± 7.9	88.3 ± 11.0	100.2 ± 7.9	88.1 ± 14.3	89.1 ± 10.6
Re	91.4 ± 7.0	87.1 ± 12.1	99.2 ± 9.7	85.4 ± 5.4	88.1 ± 6.5	113.5 ± 7.2	104.5 ± 7.9	96.8 ± 8.2
Sk	98.9 ± 14.7	87.2 ± 10.1	97.9 ± 8.9	93.3 ± 7.7	82.2 ± 11.9	114.6 ± 11.5	87.2 ± 12.6	98.2 ± 13.2
Sn	97.5 ± 6.9	90.6 ± 9.7	106.1 ± 11.5	91.3 ± 4.0	90.5 ± 4.8	108.0 ± 9.3	88.8 ± 7.4	100.7 ± 5.9
Sp	94.0 ± 2.8	84.3 ± 8.2	93.8 ± 13.1	83.9 ± 4.1	84.9 ± 8.0	89.8 ± 7.9	81.5 ± 6.7	90.0 ± 12.3
Sv	92.0 ± 6.0	89.1 ± 12.2	97.9 ± 9.5	89.9 ± 3.9	90.0 ± 6.4	105.1 ± 5.3	86.4 ± 7.2	101.7 ± 9.2
Td	88.9 ± 7.7	85.8 ± 9.6	84.1 ± 8.3	90.1 ± 4.0	91.4 ± 6.9	96.3 ± 6.0	95.7 ± 3.6	92.7 ± 4.2
7-Ly	91.3 ± 4.1	105.1 ± 8.3	77.9 ± 12.0	87.2 ± 4.8	89.3 ± 11.6	78.1 ± 9.7	80.5 ± 8.7	82.1 ± 3.3
7-Im	92.41 ± 6.7	100.5 ± 7.1	80.3 ± 13.5	100.0 ± 3.9	84.6 ± 5.3	86.7 ± 10.8	83.1 ± 8.2	85.9 ± 7.9
EmNO	76.0 ± 12.9	103.6 ± 14.5	91.8 ± 8.6	86.9 ± 9.2	92.9 ± 12.9	82.0 ± 12.1	85.2 ± 9.4	93.0 ± 13.9
ErNO	75.9 ± 6.6	109.4 ± 7.9	84.7 ± 8.3	92.8 ± 5.2	95.8 ± 11.9	80.5 ± 9.7	95.5 ± 10.2	84.8 ± 8.3
EuNO	96.4 ± 7.0	100.4 ± 7.3	91.5 ± 6.9	91.4 ± 7.0	101.6 ± 6.6	97.5 ± 10.4	104.1 ± 8.9	95.2 ± 11.8
HeNO	98.5 ± 3.8	101.0 ± 3.2	91.9 ± 4.3	97.0 ± 3.6	100.1 ± 4.4	97.9 ± 5.5	103.8 ± 5.8	97.5 ± 6.6
IcNO	99.3 ± 3.5	105.5 ± 6.5	89.7 ± 11.4	99.0 ± 2.1	105.2 ± 4.6	92.6 ± 6.8	96.4 ± 6.9	93.7 ± 11.3
ImNO	103.7 ± 13.0	100.4 ± 12.9	91.7 ± 9.2	100.0 ± 6.5	101.7 ± 7.7	91.3 ± 11.6	109.1 ± 12.7	97.9 ± 11.4
JbNO	75.4 ± 7.7	100.7 ± 7.0	74.9 ± 7.3	88.7 ± 8.0	96.3 ± 10.0	78.5 ± 9.4	104.0 ± 8.9	85.5 ± 7.5
LcNO	84.2 ± 15.4	91.8 ± 11.7	90.4 ± 9.8	94.8 ± 7.7	88.8 ± 12.6	83.2 ± 12.9	86.0 ± 13.9	80.2 ± 11.6
LyNO	98.4 ± 9.0	111.8 ± 9.5	86.0 ± 8.8	97.0 ± 4.4	109.2 ± 5.4	100.6 ± 8.5	102.6 ± 9.4	90.6 ± 9.7
McNO	83.6 ± 5.0	101.5 ± 7.8	78.5 ± 9.4	91.3 ± 5.0	103.1 ± 9.5	80.8 ± 7.5	90.4 ± 4.9	85.2 ± 8.6
ReNO	93.8 ± 8.3	100.2 ± 9.4	88.3 ± 6.9	95.5 ± 9.6	102.5 ± 15.2	94.1 ± 9.9	99.7 ± 5.8	95.9 ± 12.2
SnNO	95.7 ± 3.2	107.5 ± 4.8	80.3 ± 9.1	98.6 ± 5.9	106.8 ± 9.3	90.4 ± 12.3	94.8 ± 5.3	97.5 ± 9.6
ReNO	93.8 ± 8.3	100.2 ± 9.4	88.3 ± 6.9	95.5 ± 9.6	102.5 ± 15.2	94.1 ± 9.9	99.7 ± 5.8	95.9 ± 12.2
SnNO	95.7 ± 3.2	107.5 ± 4.8	80.3 ± 9.1	98.6 ± 5.9	106.8 ± 9.3	90.4 ± 12.3	94.8 ± 5.3	97.5 ± 9.6

**Table 6:** Accuracy, precision, stability, and LOQ of CDDP.

Compound	Accuracy (average ± RSD%)	Precision (average ± RSD%)	Stability (RSD%)	LOQ (*μ*g/kg)
Em	81.5 ± 12.0	90.0 ± 13.7	19.6	0.7
Er	85.0 ± 7.8	35.4 ± 28.3	2.0	1.5
Eu	97.7 ± 9.9	97.5 ± 5.9	8.0	0.6
He	97.1 ± 4.0	99.8 ± 2.7	4.8	0.6
Ic	94.4 ± 4.1	94.9 ± 3.1	4.3	0.7
Im	102.5 ± 8.7	102.2 ± 8.8	9.7	0.5
Jb	71.8 ± 2.7	68.9 ± 4.1	1.8	1.5
Lc	90.5 ± 12.0	99.5 ± 15.5	12.6	0.6
Ly	94.9 ± 7.3	92.9 ± 5.1	5.4	0.4
Mc	89.1 ± 5.8	90.0 ± 7.2	5.9	1.1
Re	82.0 ± 6.4	84.6 ± 4.3	7.2	3.8
Sk	95.6 ± 17.1	110.7 ± 8.1	23.9	2.0
Sn	87.0 ± 6.6	89.1 ± 3.6	4.5	3.5
Sp	74.6 ± 6.9	74.6 ± 3.4	1.6	1.2
Sv	88.4 ± 10.1	85.2 ± 3.5	2.1	2.1
Td	88.4 ± 6.6	89.1 ± 5.9	3.9	1.5
7-Ly	76.4 ± 6.8	75.7 ± 5.5	4.5	0.8
7-Im	76.2 ± 4.6	77.4 ± 5.3	8.5	0.8
EmNO	94.3 ± 11.0	86.5 ± 13.9	4.8	3.9
ErNO	91.4 ± 6.9	53.2 ± 26.9	6.9	1.3
EuNO	98.5 ± 10.5	102.7 ± 3.2	6.6	1.2
HeNO	103.6 ± 2.8	105.3 ± 2.1	2.6	1.5
IcNO	104.5 ± 2.0	103.6 ± 2.2	2.3	0.5
ImNO	104.3 ± 10.8	107.3 ± 9.5	2.9	0.6
JbNO	86.2 ± 10.5	88.8 ± 7.9	6.8	1.3
LcNO	99.2 ± 10.5	89.4 ± 13.4	1.8	0.3
LyNO	102.0 ± 5.8	98.0 ± 1.9	2.7	1.8
McNO	106.0 ± 8.7	104.7 ± 7.9	7.8	7.7
ReNO	100.5 ± 6.1	101.4 ± 4.7	3.1	3.2
SnNO	109.8 ± 6.0	112.7 ± 4.3	1.7	0.7
SpNO	80.5 ± 6.1	68.5 ± 19.3	1.2	1.5
SvNO	112.0 ± 4.7	113.3 ± 3.0	3.5	0.3

## Data Availability

Relevant research materials are kept in international Industry Center, Tasly Pharmaceutical Group Co., Ltd.
